# Optimizing Vision Transformers for Histopathology: Pretraining and Normalization in Breast Cancer Classification

**DOI:** 10.3390/jimaging10050108

**Published:** 2024-04-30

**Authors:** Giulia Lucrezia Baroni, Laura Rasotto, Kevin Roitero, Angelica Tulisso, Carla Di Loreto, Vincenzo Della Mea

**Affiliations:** 1 Department of Mathematics, Computer Science and Physics, University of Udine, 33100 Udine, Italy; giulialucrezia.baroni@gmail.com (G.L.B.); rasotto.laura@spes.uniud.it (L.R.); kevin.roitero@uniud.it (K.R.); 2 Istituto di Anatomia Patologica, Azienda Sanitaria Universitaria Friuli Centrale, 33100 Udine, Italy

**Keywords:** breast cancer, deep learning, histology, normalization, transformers

## Abstract

This paper introduces a self-attention Vision Transformer model specifically developed for classifying breast cancer in histology images. We examine various training strategies and configurations, including pretraining, dimension resizing, data augmentation and color normalization strategies, patch overlap, and patch size configurations, in order to evaluate their impact on the effectiveness of the histology image classification. Additionally, we provide evidence for the increase in effectiveness gathered through geometric and color data augmentation techniques. We primarily utilize the BACH dataset to train and validate our methods and models, but we also test them on two additional datasets, BRACS and AIDPATH, to verify their generalization capabilities. Our model, developed from a transformer pretrained on ImageNet, achieves an accuracy rate of 0.91 on the BACH dataset, 0.74 on the BRACS dataset, and 0.92 on the AIDPATH dataset. Using a model based on the prostate small and prostate medium HistoEncoder models, we achieve accuracy rates of 0.89 and 0.86, respectively. Our results suggest that pretraining on large-scale general datasets like ImageNet is advantageous. We also show the potential benefits of using domain-specific pretraining datasets, such as extensive histopathological image collections as in HistoEncoder, though not yet with clear advantages.

## 1. Introduction

According to the International Agency for Research on Cancer (https://www.iarc.who.int/cancer-type/breast-cancer/ (accessed on 25 April 2024)), in 2020, breast cancer became the most commonly diagnosed cancer type in the world, accounting for about 13% of all cancer diagnoses. Certainly, early detection and precise diagnosis are crucial for efficient treatment and improved patient outcomes. Within the medical imaging field, histopathology is fundamental to the diagnostic process and the gold standard for distinguishing between benign and malignant tissue, especially in breast cancer among patients suffering from in situ and invasive carcinoma. The development of automated methods has been a prolific area of research in recent years [1] because of the hard work required to manually analyze these images, which is time-consuming, prone to errors, and needs extensive training and domain expertise [2].

In this study, we propose a self-attention Vision Transformer (ViT) model [3] tailored for breast cancer histology image classification, which extends and refines a previous preliminary work [4]. The performance of the presented model is evaluated using different training strategies and configurations, involving pretraining, resize dimension, data augmentation, patch overlap, and patch size, to examine their impact on performance. The image set used is part of the BACH Grand Challenge on BreAst Cancer Histology images (BACH) [5], for which both baselines and the state-of-the-art are available. The proposed ViT model on the breast cancer histology image shows efficacy on the classification task regarding the experimental results obtained, and these findings can be useful for the implementation and design of future ViT-based models with comparable image classification tasks.

### Background and Related Work

Here, after an introduction on ViTs, we briefly present some examples of deep learning applications on different datasets of breast cancer histology images, mainly from the BACH dataset [5]. At the beginning, we illustrate the results achieved from the application of different Convolutional Neural Networks (CNNs) [6,7], and then we showcase the outcomes obtained using ViTs.

In computer vision, image classification is a critical task with several applications in fields such as autonomous vehicles, surveillance systems, and medical imaging. For many years, the dominant approach has been the use of CNNs to deal with image classification tasks because of their exceptional performance in capturing local patterns and hierarchical structures [8]. However, advances in deep learning have prepared the way for the rise of a new paradigm known as ViT [3] that has already achieved considerable success in image classification tasks. Several factors have contributed to defining the success of ViTs in image classification.

Firstly, the self-attention mechanism provides the model with the ability to dynamically assign weights to different parts (referred to as patches) of the image, with the enhancement of the representation of the whole context. This capability proves to be particularly advantageous in the situation where the valuable information necessary for discrimination is sprinkled in distant areas of the image. Secondly, with the aim to process larger input resolutions and deeper architectures, ViTs can be easily upsized, empowering the performance on complex datasets. Lastly, the pretraining and fine-tuning processes applied by ViTs allow for capable transfer learning, making them particularly suitable for situations with limited labeled data.

Chennamsetty et al. [9] used tree CNNs, a ResNet-101 [10], and two DenseNet-161 [11] networks, trained on different preprocessing modalities, to form an ensemble to classify histology images from the BACH dataset. The images are resized via bilinear interpolation to 224×224 pixels, then normalized to zero mean and unit standard deviation. In the training phase, the ResNet-101 and a DenseNet-161 are fine-tuned with images normalized from the breast histology data, while the other DenseNet-161 is fine-tuned with the ImageNet normalization. Finally, each model makes a prediction between the four classes for each image and a majority voting scheme is adopted to assign a final class of the input. Finally, the authors achieved an accuracy of 0.87 on the hidden test dataset of BACH.

Kwok [12] presented a model based on Inception-Resnet-v2 [13] that uses both microscopy images and Whole Slide Images (WSIs) [14,15] from BACH to train a patch classifier in two steps. Firstly, from the original images of pixel size 2048×1536, he extracts 5600 patches with a size of 1495×1495 pixels with a stride of 99 pixels, resizes them to 299×299 pixels, and uses them for fine-tuning a four-class Inception-Resnet-v2 trained on ImageNet. Some patches are also extracted from the WSIs with the same modality of the first step. This second new patch dataset is refined by rejecting images with a <5% foreground and labeled using the CNN trained on the first part. Then, 5900 patches from the top 40% incorrect predictions, that are fairly sampled from each of the four classes, are selected as hard examples for the second step. For the second step, the 5600 patch extracted in the first step and the 5900 in the second step are merged, and then the CNN is retrained. The aggregation from patch-wise predictions back onto image-wise predictions and WSI-wise heatmaps gives the results of the predictions. A normalization and a threshold are applied to the map obtained with the aim to bias the predictions more towards normal and benign, and less to in situ and invasive carcinomas. At the end, the author achieved an accuracy of 0.87 on the hidden test dataset of BACH and a score of 0.69 in Part B dedicated to WSIs.

Brancati et al. [16] used a combination of three configurations of ResNet [10], 34, 50, and 101, each trained on the BACH dataset, varying from each other in relation to the number of layers, with the use of a maximum probability rule to contrast their individual weaknesses during the testing. In particular, each image is downsampled by factor *k*, 80% of the original image size, and the input to the network is only the central patch of size m×m, where *m* is set the same as the minimum size between the width and height of the resized image. Then, the final classification is achieved with the highest class probability supplied by the three configurations. Finally, the authors achieved an accuracy of 0.86 on the hidden test dataset of BACH.

Roy et al. [17] developed a patch-based classifier (PBC) using a CNN for an automated classification of breast cancer histopathology images from the BACH dataset. This model works in two different modalities: one patch in one decision (OPOD) and all patches in one decision (APOD), and both adopted a Macenko color normalization technique [18], a patch extraction (512×512 pixels), and augmentation. On the one hand, the patch labels are predicted using the first mode and the final class label is obtained from the scores returned by a softmax classifier. On the other hand, the label for the entire image is determined using a majority voting scheme with the second mode. In addition, the images are classified not only into four classes as requested by the challenge but also in two classes. The results of this research show that the OPOD model accomplishes accuracy rates of 0.77 and 0.85 for multi-class and for binary histopathological patch-wise classification, respectively, while APOD demonstrates accuracy rates of 0.90 and 0.92 for multi-class and binary image-wise classification, respectively. Finally, the authors achieved an accuracy of 0.87 on the hidden test dataset of BACH.

Recently, due to the rise of Transformers [19] in natural language processing in 2017 and their application in images with the birth of ViTs [3] in 2020, the medical imaging field has also shown an interest in the exploration and use of Transformer-based techniques for different tasks and challenges [19,20]. Thus far, there have only been a few studies that have investigated the application of ViTs in the breast cancer domain with detection, segmentation, and classification [21]. Moreover, as underlined in recent research articles [21,22], the capabilities of ViTs in the medical field are not already known and have not been explored completely.

Wang et al. [22] proposed a semi-supervised learning procedure based on the ViT with not only an adaptive token sampling (ATS) technique to advantageously sample the most significant tokens from the input images, but also a consistency training (CT) strategy that combines supervised and unsupervised learning with image augmentation. Thus, ATS-ViT is the core model used in the training process that consists of two parts: the first one, the supervised training, improves the predictive ability of the model, while the second one, the consistency training, enhances its generalization. These two parts are then unified via an end-to-end training procedure. The authors evaluated the model using two different datasets: one with breast ultrasound images (BUSI) and the other with breast histopathology images (BreakHis [23]). The best approach, CT + ViT + ATS, achieved an average test accuracy of 0.98 on the BreakHis dataset, outperforming the CNN baselines.

Tummala et al. [24] presented a variant of ViT, called Swin Transformer (SwinT) [25], that works on the concept of non-overlapping shifted windows. Using the BreakHis dataset, the study evaluates the results of an ensemble of four SwinT models not only for a binary classification (benign/malignant) but also for an eight-subtype classification (four benign and four malignant). The unique aspect of SwinT is its employing of shifted windows arranged in a non-overlapping fashion. This upgraded ViT version employs a hierarchical structure, offering linear complexity and scalability for window-based self-attention computations. The shifted window approach in SwinT boosts efficiency by limiting self-attention calculations to local, non-overlapping windows, thereby facilitating connections across different windows. The ensemble of SwinTs, which includes the tiny, small, base, and large variants, achieved an average test accuracy of 0.99 for the two-class classification and of 0.96 for the eight-class classification.

Alotaibi et al. [26] presented an ensemble model that merges two pretrained models, namely ViT and Data-Efficient Image Transformer (DeiT) [27], with the aim to classify breast cancer histopathology images into eight classes using the BreakHis public dataset. While the ViT model follows a similar operational approach to earlier studies, its novelty lies in the addition of DeiT, which uses an extra input called the distillation token. The learning process of the distillation token involves backpropagation, where it interacts with classes and patch tokens through the self-attention layers. Thus, DeiT introduces enhancements over previous ViT models, bringing a new level of performance and capabilities to the architecture. Both models are pretrained on ImageNet, utilizing an input size of 224×224 and a batch size of 16. Then, a soft voting technique is used to obtain the highest probability value from this ensemble model. The experimental results presented an accuracy of 0.98.

He et al. [28] depicted the exploration of histopathological image color deconvolution in conjunction with deep learning models. The researchers proposed the Deconv-Transformer (DecT) model for the classification of histopathological images of breast cancer using three datasets: the BreakHis dataset, BACH dataset, and UC dataset. The proposed DecT model predominantly relies on the Transformer architecture instead of convolution layers, aiming to align more effectively with color deconvolution processes. Then, the training process for the DecT model and its variants is divided into two stages, facilitating the discovery of more optimal parameters for the deconvolution layer. Experimental findings indicate that this approach integrates image information from both RGB and HED color spaces, surpassing the ViT model and various traditional CNN models in terms of classification accuracy. Some color data augmentation is applied to attenuate overfitting during mode training. The final results showed that the DecT model achieved an average accuracy of 0.93 on the BreakHis dataset, 0.79 on the BACH dataset, and 0.81 on the UC datasets.

Sriwastawa and Arul Jothi [29] presented a wide comparison between performances of several newer models of the ViT, in particular the Pooling-based Vision Transformer (PiT) [30], Convolutional Vision Transformer (CvT) [31], CrossFormer [32], CrossViT [33], NesT [34], MaxViT [35], and Separable Vision Transformer (SepViT) [36], with the aim to show the enhancement of the accuracy and generalization ability of ViT. They employed the BreakHis and IDC datasets [37,38]. In particular, all chosen models were independently trained from scratch on the BreakHis and IDC datasets, and the models trained on BreakHis were fine-tuned using IDC. MaxViT seems to be the best transformer-based classifier, achieving a test accuracy of 0.916 on BreakHis, 0.92 on IDC, and 0.92 when pretrained on BreakHis and then fine-tuned on IDC, directly followed by NesT and CvT. Despite this, according to the researchers, none of the models manifest a significantly improved performance compared to existing works.

In Table 1 we summarize the models we take into account in the selected studies in which breast cancer is classified.

Regarding the CNN models, as Chennamsetty et al. [9] report in their research, the performance of CNN is dependent on the network architecture and number of training instances, and also on the data normalization scheme. Up to now, there is neither a single architecture nor a preprocessing modality that promises to achieve the best performance. For that reason, the best way to obtain a good result seems to create an ensemble of networks with different preprocessing regimes, as demonstrated by all the studies considered. Therefore, the main drawback is the computationally more expensive approach of involving an ensemble of models. For example, as Brancati et al. [16] explain in their study, ResNet was adopted instead of other deep network architectures because it has a small number of parameters and it needs a relatively low complexity compared to other models.

Regarding the ViT models, not only the forming of an ensemble of networks with different preprocessing modalities is experimented on, as shown by Wang et al. [22] and Tummala et al. [24], but also only one type of network is investigated, as shown by He et al. [28] and Sriwastawa and Arul Jothi [29]. In those cases, they utilize the BreakHis dataset for two- and eight-class classifications, which are not directly comparable with the BACH dataset we employ containing four classes. The experiment conducted by He et al. [28], which was the only one to utilize the same dataset as us, achieved an accuracy rate of 0.79. The experimental results of Wang et al. [22], who used an ensemble of CT + ViT + ATS, show that the original ViT model does not present superior performance compared to its CNN competitors, and also Sriwastawa and Arul Jothi [29], even with the use of different single ViTs, explain that none of the models reveal a significantly improved performance compared to existing works.

Until now, only a limited number of studies have explored the use of ViTs in the field of breast cancer histology image for classification [21,40]. Thus, our study showcases an extensive and perceptive exploration into the use of ViT models for this purpose. We aim to establish a robust foundation highlighting the advantages of employing ViTs in this field, specifically by examining the impact of pretraining, augmentation methods, patch configurations, and also some integration of domain-specific tools. First of all, unlike the examples extensively reported in Table 1 where ensemble models are predominantly used, we improve the model’s classification capacity and subsequently its generalization with a limited computational cost and without any ensemble of models. In fact, we use only a ViT-based model, without any particular parameters being tuned or featuring engineering. Secondly, we apply some basic pretraining strategies, such as geometric and color data augmentation and Macenko’s color normalization, that are found to be sufficient to increase the accuracy rate. Then, our model is shown to work in a good manner without any other preprocessing of the images, unlike the common practice in the medical field with CNNs extracting some patches with or without overlap, as demonstrated by Kwok [12] and Brancati et al. [16]. In addition, we fine-tuned a custom neural network leveraging HistoEncoder [41], a tool designed for digital pathology, not only to find the potentiality of using a domain-specific training in histology images, but also to compare the results of a model from a pretraining with prostate cancer images to a fine-tuning with breast cancer ones. Finally, our best ViT model is tested on two other datasets, the BRACS [42] and AIDPATH (https://aidpath.eu (accessed on 25 April 2024)), with the aim of studying the model’s generalization deeply.

We focus on the following Research Questions (RQs):

**RQ1**: Is it possible to fine-tune a ViT model for breast cancer classification?
**RQ2**: What are the effects of using pretraining strategies, such as data augmentation or normalization?
**RQ3**: Is it possible to generalize our results to other datasets?

The rest of this paper is organized as follows. In Section 2, we firstly present the main dataset we use, BACH, and the other two datasets, BRACS and AIDPATH, which we add to evaluate our model. Then, we describe the general characteristics of our ViT model and its configurations. In Section 3, we present the results of our study, including an in-depth analysis of our pretraining outcomes, the evaluation of robustness through k-fold cross-validation, and an assessment of the model’s generalization capability. In Section 4, we discuss our results and key findings. Finally, in Section 5, we draw our conclusions.

## 2. Materials and Methods

### 2.1. Datasets

The developed models were trained on a single dataset, namely BACH, and then tested not only on the same dataset but also on two further datasets to study their generalizability capabilities. They are described in the next sections.

#### 2.1.1. BACH: Grand Challenge on BreAst Cancer Histology Images

In 2018, for the promotion of the development of automated breast cancer detection and diagnosis methods, Grand Challenge on BreAst Cancer Histology images (BACH) (https://iciar2018-challenge.grand-challenge.org (accessed on 25 April 2024)) [5] was organized in conjunction with the 15th International Conference on Image Analysis and Recognition (ICIAR 2018), obtaining classification and localization of relevant histopathology classes in medical imaging, both microscopy and WSIs, from an annotated dataset, specifically compiled and made publicly available for the challenge. The BACH challenge was divided into Part A and Part B. The first one comprises hematoxylin and eosin (H&E)-stained breast histology microscopy images split in four classes: (1) normal, (2) benign, (3) in situ carcinoma, and (4) invasive carcinoma. The second one is composed of pixel-wise segmented WSIs with the same four classes. The post-challenge submissions, which required an evaluation of the results, are open only on Part A images and tasks. Therefore, in this paper, we participate in a single part of the challenge: we focus on Part A with an experiment integrating the image set from Part B. Additionally, this means that to evaluate the results, we can refer to the metrics provided by the post-challenge submission, i.e., only the accuracy rate.

**Figure 1:**
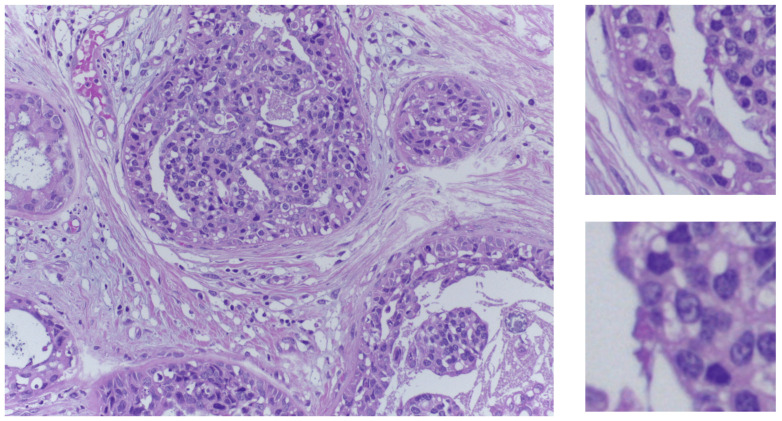
On the left side, an in situ carcinoma image from the training set. On the right side, two sample patches in the two sizes used in our experiments.

The Part A dataset comprises 400 training images, distributed equally for the four classes and 100 test images. For the acquisition of the images, a Leica DM 2000 LED microscope and a Leica ICC50 HD camera are used. All patients originate from the Porto and Castelo Branco regions in Portugal; cases are from Ipatimup Diagnostics and come from three different hospitals. The given images are in RGB TIFF format and have a size of 2048×1536 pixels with a pixel scale of 0.42 μm × 0.42 μm. An example image is depicted in Figure 1 that shows two patch sizes we tested in our experiments. For the training set, a partial patient-wise distribution of the images is given to participants, while for the test set, images are collected from a completely distinct set of patients, ensuring a fair evaluation of the methods.

The best two teams that took part in the challenge obtained the same accuracy of 0.87 [5]. Then, for post-challenge evaluations, the submission was re-opened and, in this phase, some slightly better results were achieved, also exploiting data from other datasets. For instance, Yao et al. [39] with a complex ensemble of CNN and RNN on the BACH test dataset reached an accuracy of 0.92.

#### 2.1.2. BRACS: BReAst Carcinoma Subtyping Dataset

The BReAst Carcinoma Subtyping (BRACS) dataset (https://www.bracs.icar.cnr.it (accessed on 25 April 2024)) comprises a large collection of annotated histopathology images stained with H&E and used to study breast carcinoma [42]. The dataset was created through a collaboration between the IRCCS Fondazione Pascale, the Institute for High Performance Computing and Networking (ICAR) of the National Research Council (CNR), and IBM Research-Zurich, aiming to develop methodologies for identifying atypical tumors in breast cancer pathology via automated analysis of histological images. BRACS is particularly valuable for researchers looking to test and compare automated detection and classification strategies for breast tumors, as it includes a range of lesions typically found in breast tissue. These lesions belong to different kinds, such as Pathological Benign (PB), Usual Ductal Hyperplasia (UDH), Flat Epithelial Atypia (PEA), Atypical Ductal Hyperplasia (ADH), Ductal Carcinoma in situ (DCIS), and Invasive Carcinoma (IC). The dataset also includes images representing normal (N) tissue samples, which are glandular tissue samples without any lesions. ADH and FEA are considered intraductal proliferative lesions that are associated with an increased risk of cancer. They are not “precursors” of cancer but lesions that predispose to an increased risk of cancer. Even in the WHO classification they are classified separately as intraductal proliferative lesions [43,44].

**Figure 2:**
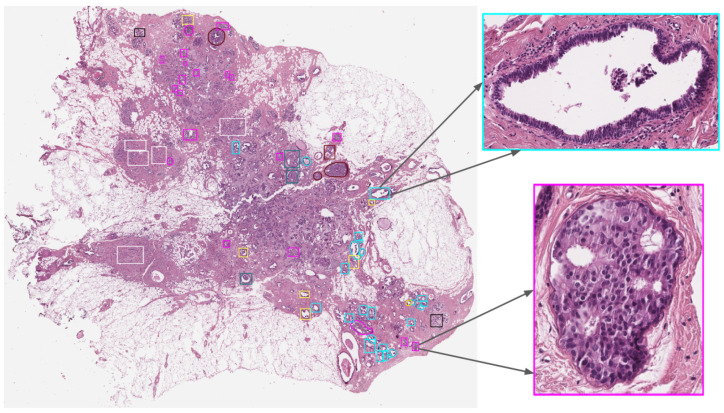
Example of a BRACS WSI with annotations and its detailed RoI adapted from Brancati et al. [42].

The BRACS dataset contains 547 WSIs collected from 189 patients and 4537 Regions of Interest (RoIs) extracted from 387 WSIs collected from 151 patients. One example of WSI from this dataset is shown in Figure 2. WSIs of H&E-stained breast tissues were generated by using an Aperio AT2 scanner at 0.25 μm/pixel for 40× resolution.

#### 2.1.3. AIDPATH

As part of the MSCA European Project AIDPATH (https://aidpath.eu (accessed on 25 April 2024)), a dataset consisting of 50 WSIs of an invasive tumor acquired at 40× magnification has been collected and made available. These images are only weakly annotated, so their use for testing is limited to the capability of correctly classifying invasive tumors.

### 2.2. Overview of ViT Approach

**Figure 3:**
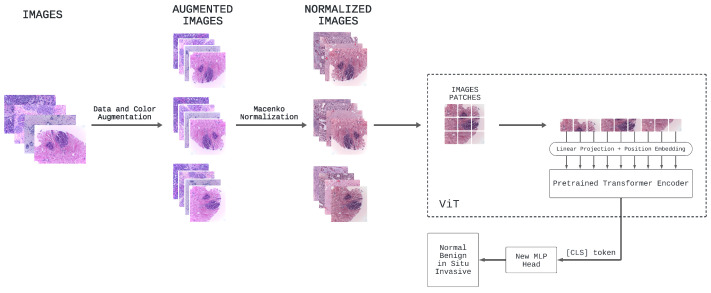
Overview of the proposed self-attention ViT model for classifying breast cancer in histopathology images.

In this research, the ViT model serves as the foundational architecture for all our experiments, owing to its established success in diverse computer vision tasks, especially image classification [8,45,46]. We present in Figure 3 a tailored, customized self-attention ViT specifically designed for classifying breast cancer in histology images.

In the preprocessing phase, various data and color augmentations are applied to all images in Part A of the BACH dataset. Specifically, to the initial 400 images of the dataset under consideration, additional images are generated through different samplings of data and color augmentation techniques of the original dataset, including rotation, scaling, flipping, and color jittering, in order to introduce as much variation as possible. Subsequently, Macenko’s color normalization is applied to all of these images.

Consequently, all the breast cancer histology images, which now create a larger dataset, are used to fine-tune the original ViT model [3] pretrained on ImageNet-21k, namely google/vit-base-patch16-224-in21k. In this specific task with this pretrained ViT based on the original one, the process of fine-tuning enables leveraging the knowledge learned from a large dataset (ImageNet-21k) to improve performance on a specific task (the classification of breast cancer images) using a smaller dataset (the BACH dataset with data and color augmentations).

In detail, the core of our ViT architecture comprises a stack of identical transformer layers [19]: each layer includes a multi-head self-attention mechanism followed by a position-wise feedforward network. The self-attention mechanism computes attention scores among each patch and all other patches in the input sequence, facilitating the model’s ability to capture both local and global contextual information. This capability enables accurate and robust predictions of breast cancer sub-types. After processing the fixed-size image patches through these transformer layers, the pretrained prediction head is removed and replaced with a new feedforward layer consisting of four outputs, corresponding to the number of classes in the breast cancer classification task. This new feedforward layer, along with the [CLS] token, enables the model to adapt to the specific classification task by employing a classification head in the form of a projection applied to the final hidden state of the [CLS] token. The responsibility of this classification head is to predict the logits associated with different breast cancer sub-types, which represent the considered classes. It consists of a linear layer that performs the necessary projection, followed by a softmax activation function, yielding a probability distribution across the four selected classes.

### 2.3. Model Configurations

In this study, we investigate various training strategies and configurations for our self-attention ViT model to assess their impact on the performance of breast cancer histology image classification.

- *Pretraining Strategy*: Distinct strategies in our experimentation concerning the use of pretrained weights [47] are explored. The first strategy entails the utilization of a ViT model that has undergone prior training on a substantial dataset. Specifically, we make use of the google/vit-base-patch16-224-in21k model (https://huggingface.co/google/vit-base-patch16-224-in21k (accessed on 25 April 2024)) that underwent pretraining on ImageNet-21k, comprising 14 million images and approximately 22,000 classes, at a resolution of 224×224. This approach is motivated by the concept of transfer learning [48,49], which capitalizes on the knowledge acquired from the source domain to enhance performance in the target domain. On the other hand, the second strategy entails using the same ViT architecture but initializing the model’s weights randomly. This strategy, in essence, involves training the model from scratch.
- *Resize Dimension*: An examination is undertaken into the consequences of resizing the input images to a standardized dimension of 224×224 pixels, a prevalent practice in image classification tasks. Additionally, we explore the implications of abstaining from resizing the input images, allowing the model to operate with their original dimensions.
- *Data Augmentation*: The application of geometric data augmentation techniques, including rotation, scaling, and flipping, to enhance the model’s generalization capabilities is investigated. Additionally, color data augmentation methods such as color jittering are explored. These modifications to our input images serve to artificially expand and diversify our dataset, thereby equipping the model with more robust and invariant features. We also consider an experimental scenario where no data augmentation is employed to assess the model’s performance in the absence of the additional diversity introduced by these augmentation processes. Furthermore, we shift our focus to the Part B dataset, incorporating tiles derived from WSIs. A single WSI can encompass multiple regions categorized as normal, benign, in situ carcinoma, and invasive carcinoma.
- *Color Normalization*: The importance of using Macenko’s color normalization [18,50] lies in its ability to standardize the appearance of digital histopathology images across different conditions. Images can vary significantly in color due to differences in staining processes, scanners, and lighting conditions. Macenko’s method addresses this issue by normalizing the colors in the images, ensuring that the tissue samples appear consistent across different images. This consistency is crucial for accurate diagnosis and research, as it allows for more reliable comparison and analysis of tissue samples. The method works by modeling the stain color and intensity distribution, then adjusting the images to fit a standard model. This not only aids pathologists in making more accurate diagnoses but also improves the efficacy of automated image analysis systems, which play an increasingly important role in histopathology.
- *Tile Overlap*: This refers to the number of pixels shared in common between two adjacent tiles. Increasing the overlap can potentially enhance the model’s performance by facilitating better integration of information across tiles. However, this improvement comes at the cost of increased computational demands. Therefore, our objective is to determine the optimal overlap that strikes a balance between model performance and computational efficiency.
- *Tile Patch Size*: The patch size plays a central role in defining the level of localized information the model can gather from each patch. Larger patches empower the model to capture intricate local nuances, while smaller patches facilitate a broader contextual perspective. However, opting for larger patches amplifies the computational workload due to the increased number of pixels within each patch. Hence, our aim is to identify the optimal patch size that effectively balances the trade-off between capturing local and global information while preserving computational efficiency.

## 3. Results

This study presents a detailed evaluation of various ViT models for breast cancer histology image classification, as shown in Table 2. Different ViT models are evaluated considering a range of parameters such as pretraining strategies, data augmentation methods, tile overlap, patch size, and image resizing techniques, with accuracy as the primary performance metric.

- Initially, the baseline ViT model employed 512 × 512 tiles with a 256 overlap and a 32 × 32 patch size, without any image resizing or pretraining, leading to an accuracy of 0.53. This suggests the chosen patch size may not adequately capture sufficient local detail for accurate predictions.
- Subsequently, the model was modified to use 512 × 512 tiles resized to 224 × 224, fine-tuned exclusively on the breast cancer dataset, with a 16 × 16 patch size and a 256 tile overlap, resulting in a similar accuracy score of 0.53.
- Enhancements were made in the next iteration where the base ViT model, pretrained on ImageNet and fine-tuned on the breast cancer dataset, used 512 × 512 tiles resized to 224 × 224 and a 256 tile overlap with an improved accuracy score of 0.84, emphasizing the significance of pretraining on extensive datasets.
- In the fourth variation, tile overlap was deleted. The images of 2048 × 1536 pixels were resized to 224 × 224, achieving an accuracy of 0.84. This indicates that increasing tile overlap does not necessarily improve performance, and may in fact introduce computational inefficiency and redundancy.
- Further improvements were observed in the fifth iteration, using the base ViT model pretrained on ImageNet and fine-tuned on the breast cancer dataset, with a 16 × 16 patch size and no tile overlap. The incorporation of geometric and color data augmentation techniques resulted in an enhanced accuracy of 0.90, demonstrating the effectiveness of these augmentations in model generalization.
- The final iteration involved pretraining the same ViT model on ImageNet, followed by fine-tuning on a comprehensive breast cancer dataset comprising both image-wise labeled histology images (Part A) and pixel-wise labeled cropped tiles from WSIs (Part B). The images in Part B were rescaled to match the pixel scale of Part A. Macenko color normalization was applied to all images to minimize stain variability, achieving the highest accuracy score of 0.91.

### 3.1. Pretraining Outcomes: A Detailed Assessment of HistoEncoder Strategies

HistoEncoder [41] is a Python package and a Command Line Interface (CLI) tool designed for digital pathology. Its primary function is to extract and cluster features from histological slide images, aiding in the analysis of these images. It operates through a self-supervised pretraining process. During this phase, the models learn to produce similar features for images that exhibit similar histological patterns. HistoEncoder was developed using comprehensive foundation models and it was trained on significant prostate tissue data using LUMI, one of Europe’s leading supercomputers, generating two models: prostate_small and prostate_medium. Both models are designed to extract and cluster features from histological slide images.

In this study, we develop a custom neural network leveraging HistoEncoder. The network architecture comprises an encoder, created via HistoEncoder, which processes the input images to extract relevant features. Additionally, a linear layer is integrated, mapping these features to class scores, which is a crucial step in the classification process. This design facilitates the model’s learning from input images, enabling precise classification into distinct categories, thereby demonstrating its effectiveness in handling specific histopathological image data.

The best achieved accuracy rates for the models are 0.89 and 0.86 for the prostate small and prostate medium models, respectively, as shown in Table 3. The achieved results, although promising, still fall short of the higher accuracy rates obtained using google/vit-base-patch16-224-in21k, the pretrained model from Hugging Face. This model reports accuracy rates of 0.90 and 0.91, respectively, when enhanced with data augmentation techniques and Macenko normalization, along with the utilization of dataset Part B. These advanced techniques contribute to the model’s superior performance in histopathological image classification tasks. On the other side, the sub-optimal performance obtained using HistoEncoder might be in part due to the different organ used in training HistoEncoder.

### 3.2. The Robustness through the Use of K-Fold Cross-Validation

K-fold cross-validation stands as a critical method in the fields of statistics and machine learning, serving as a key tool for assessing the performance of models. Choosing a 5-fold cross-validation involves dividing our dataset into five equal parts where each fold should ideally represent the overall data distribution. For each round of validation, one of the five folds is used as the validation set. The decision to employ 5-fold cross-validation is strategically made to strike a balance between bias and variance, two crucial aspects in model evaluation. This approach offers a well-rounded assessment, ensuring neither overfitting nor oversimplification of the model. In Table 4, after running all five rounds, we show the performance measures, such as accuracy, precision, recall, and F1-Score, from each round are averaged to give an overall performance metric for the model.

**Figure 4:**
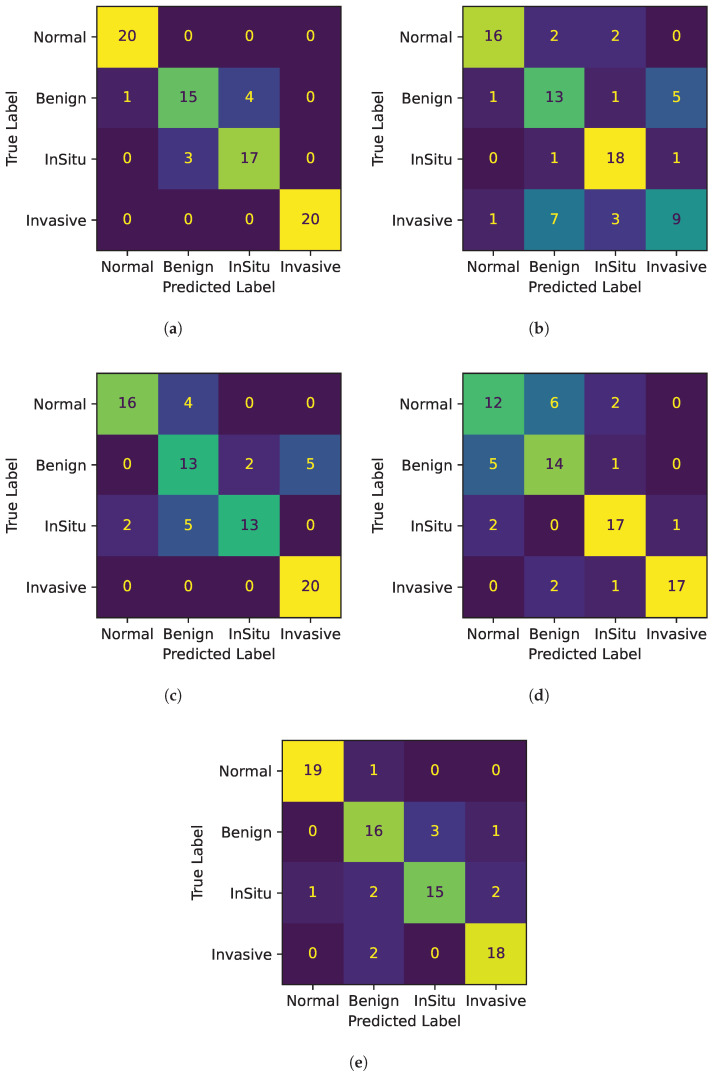
Confusion matrices for all 5 folds. (**a**) Confusion Matrix Fold 1. (**b**) Confusion Matrix Fold 2. (**c**) Confusion Matrix Fold 3. (**d**) Confusion Matrix Fold 4. (**e**) Confusion Matrix Fold 5.

Table 4 is divided into four classes: normal, benign, in situ, and invasive. For each class, the table lists performance metrics such as accuracy, precision, recall, and the F1-Score for each fold. The table presents the data obtained from the 5-fold cross-validation with a subdivision of evaluation metrics adopted, highlighting their behavior in each class, as well as the mean and the standard deviation. In general, it can be seen that the normal and invasive classes are better predicted than the benign class, while the in situ class is predicted as slightly worse than the first two. Some considerations are reported below:

- *Accuracy*: Confusion matrices for all 5-fold cross-validations are depicted in Figure 4. Each figure shows a heatmap obtained from a fold. In the x-axis the predicted labels between normal, benign, in situ, and invasive are represented, while the y-axis presents the true labels. In other words, each row of the matrix illustrates the occurrences in the true class, while each column represents the occurrences in the predicted class. The elements on the main diagonal, from top left to bottom right, show the number of samples that are correctly classified. For instance, in the heatmap of Fold 1, the samples belonging to the normal and invasive classes, and in the heatmap of Fold 3, the samples belonging to the invasive class, are all correctly predicted. Instead, elements outside the main diagonal represent misclassifications. The final accuracy score for each fold is 0.90, 0.70, 0.78, 0.75, and 0.85 respectively. It is clear that the model’s accuracy varies significantly across folds, particularly for the invasive class, where the accuracy ranges from 0.45 (Fold 2) to 1.00 (Folds 1 and 3).
- *Precision*: The model’s precision is generally quite high, with the normal class showing the highest mean precision at 0.86. However, the invasive class again shows a high variation in precision across folds at 0.15.
- *Recall*: For the in situ class, the recall is quite stable, with a mean of 0.80 and a low standard deviation of 0.10, suggesting consistent performance in identifying this class across different subsets of data.
- *F1-Score*: The F1-Scores in the table generally follow the trends of precision and recall, with the normal class having the highest mean F1-Score of 0.85, while the benign class has the lowest at 0.69.

Table 4 also indicates the mean and standard deviation. By looking at the average of the performance metrics, the invasive class has the highest average accuracy, while the benign class has the lowest average accuracy. In particular, the normal and the invasive classes show high mean values across all metrics with relatively low standard deviations for the first class, indicating that the model performs well and in a consistent manner for this class. This is desirable in a predictive model, as it suggests that the model’s performance is reliable and not heavily dependent on the specific data it was tested on. In contrast, the performance is less consistent when it comes to the other classes, particularly the invasive class, where it is notable that it has a higher variance than other classes, indicating greater variability in model performance for this class. This might suggest that this class might be more difficult to distinguish than the other classes.

Despite the higher variance, the model seems to be quite effective in distinguishing the invasive class from the other classes, as the accuracy and precision metrics are higher. This may suggest that the model has learned to recognize some distinguishing features of the invasive class, although its performance may vary considerably across cross-validation folds. This behavior can spotlight that this class may have more pronounced distinguishing features than other classes in the dataset that are better for classifying it.

**Figure 5:**
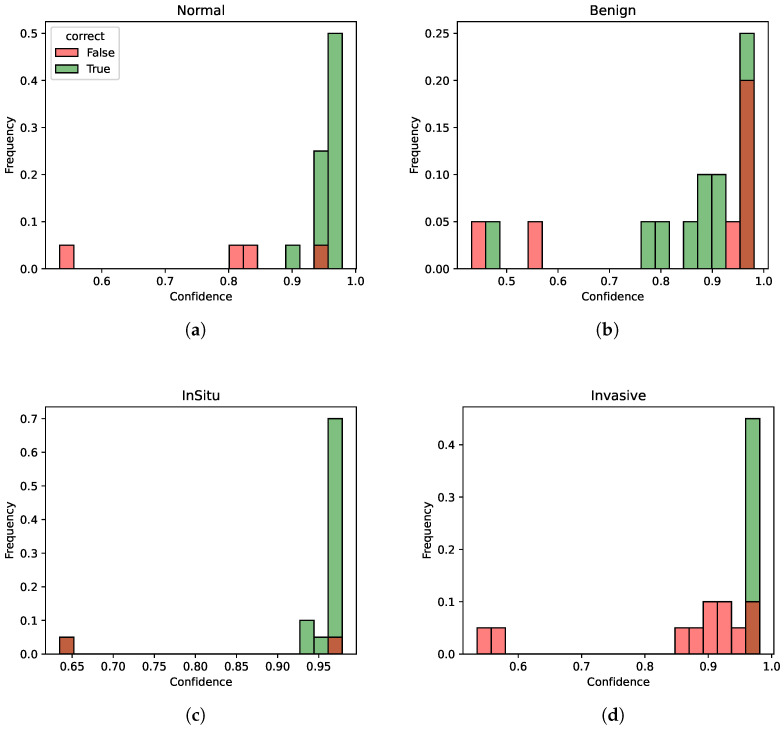
Probability distributions for Fold 2. (**a**) Probability distribution for class normal. (**b**) Probability distribution for class benign. (**c**) Probability distribution for class in situ. (**d**) Probability distribution for class invasive.

Overall, Fold 1 and Fold 5 perform better than the others as we can see by the results of the different metrics. In particular, we investigate the fold with the least performance to highlight model weaknesses, that is, number 2. In Figure 5, the distribution of the predicted probabilities for each class of Fold 2 is presented, divided between correct and incorrect predictions. Thus, each figure shows the probability distribution of the normal, benign, in situ, and invasive class. For each class, both correctly and incorrectly predicted samples for the current class are calculated. Then, two histograms are created: one for correct predictions with the color green and one for incorrect predictions with the color red. Both histograms show the distribution of the predicted probabilities for the current class.

Although the different metrics are rather low for this fold (especially for the benign and invasive classes), indicating potential weaknesses in certain aspects of the model’s performance, the model still manages to discriminate quite well between the different classes in terms of confidence scores, as the confidence for the correctly identified instances is generally higher than the one for incorrectly classified instances. Thus, this model indicates an overall good degree of consistency. In other words, despite the performance not always being optimal on individual metrics, the model demonstrates a good capability to understand underlying patterns and distinctions within the data in terms of confidence.

### 3.3. Model Generalization

Our best ViT model was subjected to testing on the BRACS dataset [42]. The BRACS dataset, designed specifically for the study of breast cancer histopathology, offers a rich and varied source of data for such a validation. By testing the ViT on this dataset, we can evaluate the model’s capacity to generalize and accurately classify histopathology images of breast carcinoma into various sub-types. Table 5 shows the confusion matrix for this test. Since the classes on BRACS are more detailed than in BACH, we evidentiated in bold the diagonal values of the matrix.

For normal tissue (0-N), the model shows a high accuracy with 0.85. Within the benign classification, the values are 0.36 for Pathological Benign (1-PB) and 0.20 for Usual Ductal Hyperplasia (2-UDH). For in situ, corresponding to Ductal Carcinoma in situ (5-DCIS), the model reaches its peak performance at 0.88, the same as in the invasive class, represented by invasive carcinoma (6-IC). The other two classes, Flat Epithelial Atypia (3-FEA) and Atypical Ductal Hyperplasia (4-ADH), have no direct correspondence to any of the BACH classes, and are reported for completeness.

The model underwent further evaluation on the AIDPATH dataset. The achieved accuracy in binary classification (presence of tumor or not) is 0.92. This high level of accuracy underscores the model’s robustness in identifying invasive tumors, validating its potential applicability in clinical diagnostic settings, for example, to screen or prioritize slides to be submitted for pathologist evaluation.

## 4. Discussion

Our research highlights the potential benefits of leveraging self-attention mechanisms through the ViT model in the challenging task of breast cancer histology image classification. We find that pretraining significantly improves the model’s performance, as evidenced by the notable decrease in accuracy upon removing the pretraining task. This underscores the advantage of transferring knowledge from extensive, general-purpose datasets to specific medical imaging tasks, thereby potentially enhancing the model’s generalization capacity and resilience to dataset biases.

In this study, we specifically directed our attention to Part A for post-challenge submission and we made a strategic decision to exclusively utilize the challenge dataset, a choice that aligns closely with our research objectives. This focus on the challenge dataset specifically aims to deeply understand its nuances, especially in relation to breast cancer histology images. Such a stringent focus ensures that our research remains highly relevant and directly applicable to the unique conditions and objectives of the challenge.

Regarding our choice of methodology, we deliberately opted for models based on ViTs rather than the conventional CNNs. This choice stems from the common training practices for ViT models, which are usually based on generic images and not specifically on medical images such as histopathology slides. With the growing prevalence of ViTs and transformer technology across various tech domains, our study is designed to assess their performance in the context of histopathology image processing and analysis, a domain where evidence is less substantial [21,22]. Through this study, we aim to reveal a new application and an understanding of ViTs, thereby expanding their usability in medical imaging fields, an area in which they have not yet been traditionally utilized.

To assess the performance of our self-attention ViT model for breast cancer histology image classification, we submitted our predictions to the challenge submission platform for evaluation. Since we lack access to the labels for the test dataset, this submission falls under the category of a full-blind submission, meaning we have no prior knowledge of the correctness of our predictions. The only feedback we receive from the submission system is a single accuracy score, so it is the official performance criterion of the challenge organizers. Accuracy is a widely utilized metric in classification tasks because it offers a straightforward and easily understandable measure of our model’s effectiveness in the given task. However, it is important to acknowledge that accuracy may not always be the most suitable metric for assessing model performance, especially in cases of imbalanced datasets where it can favor the majority class. In such instances, alternative metrics like precision, recall, F1-score, and AUC-ROC may provide a more comprehensive evaluation of the model’s performance. Despite these potential limitations, in the specific context of the dataset employed in this challenge, we are constrained by relying exclusively on the accuracy metric to measure our model’s efficacy. This limitation primarily arises from our inability to access the actual labels of the images. Consequently, in our subsequent analysis, we focus on the accuracy scores obtained from the test dataset to gain valuable insights into our model’s performance under diverse scenarios, including the investigation of various hyperparameters, the adoption of diverse data augmentation methods, and the implementation of distinct preprocessing strategies outlined earlier.

The achieved accuracy of 0.91 surpasses the top results of the BACH challenge (accuracy: 0.87), albeit marginally trailing behind the best post-challenge performance (accuracy: 0.92) [39]. Notably, the latter achievement involves an ensemble of five models (comprising various CNNs and RNN [51]), representing a computationally more intensive approach compared to ours.

Our investigation into the impact of geometric and color data augmentation yields significant insights. Disabling augmentation leads to a notable accuracy decline from 0.90 to 0.84, indicating that such augmentation techniques enhance the model’s ability to generalize to unseen images, likely by improving its resilience to minor data variations. Additionally, the utilization of tiles extracted from entire digital slides from set Part B, coupled with Macenko’s color normalization method, results in a commendable accuracy score of 0.91 on the test dataset.

Furthermore, our study delves into the effects of patch size and overlap on model performance. We find that a patch size of 16 × 16 without overlap produces optimal results, underscoring the importance of careful patch configuration in ViT models. Interestingly, larger tile overlap and larger patch sizes appear to offer no discernible benefits, potentially due to information redundancy and inadequate local information, respectively. Notably, tiling emerges as a parameter that negatively impacts accuracy. The poorest results are observed when images are split into tiles, regardless of subsequent resizing. This unexpected outcome is likely attributed to the fact that while each original image represents a specific class, it does not exclusively contain tissue from that class, particularly concerning pathological tissues. Consequently, many tiles lack specificity.

In the experiment using HistoEncoder, although we achieve promising results with accuracy rates of 0.89 and 0.86 for the prostate_small and prostate_medium models, respectively, these accuracies are slightly lower than those obtained with the pretrained model from Hugging Face’s repository, which has accuracy rates of 0.90 and 0.91, respectively. This indicates the effectiveness of leveraging large-scale, general-purpose datasets to enhance model performance.

Testing the ViT model on the BRACS dataset, our results indicate a high degree of model accuracy in classifying normal tissue and a notable performance in distinguishing between IC and DCIS, with a peak accuracy of 0.88 in both categories. However, the significantly lower accuracy for PB and UDH suggests areas for further model refinement, particularly in differentiating between benign conditions and early-stage cancerous lesions.

The additional evaluation on the AIDPATH dataset, focusing on invasive tumor detection and achieving an accuracy rate of 0.92, further suggests the model’s robustness and its potentiality for clinical application, although limited by the absence of non-cancer samples. This might suggest the model’s capacity to effectively identify invasive tumor patterns, which in turn might be the basis for screening slides and prioritizing those in need for quick pathologist examination.

These insights underline the potential of ViT models, coupled with strategic dataset utilization and augmentation techniques, to enhance the accuracy and efficiency of histopathology image analysis.

## 5. Conclusions

Our research demonstrates a comprehensive and insightful exploration into the application of ViT models for breast cancer histology image classification, primarily utilizing the BACH dataset. The detailed examination of pretraining effects, resize dimension, augmentation techniques, color normalization, and patch configurations, and the integration of domain-specific tools like HistoEncoder, provides a solid foundation for the advantages of employing ViT in this domain. The findings not only contribute to the ongoing discourse on leveraging advanced machine learning models for medical imaging but also highlight the importance of dataset selection and preparation in achieving high model performance.

We provide the following answers to the RQ:

**RQ1**: To answer RQ1, we can say that it is possible to fine-tune a Vision Transformer (ViT) model for breast cancer classification; in particular, adapting the chosen pretrained ViT model google/vit-base-patch16-224-in21k, which has learned general features from a large dataset, to the specific task of breast cancer classification using the histology images of the BACH dataset. Therefore, fine-tuning a ViT model for breast cancer classification is not only possible but also a practical and effective approach to leveraging the representation learning capabilities of pretrained models for specific medical imaging tasks.
**RQ2**: To address RQ2, we can assert that our results indicate that pretraining ViT models on ImageNet, coupled with geometric and color data augmentation, significantly enhances performance in breast cancer histology image classification tasks. Optimal results are achieved with a 16 × 16 patch size and no tile overlap. These findings offer crucial insights for the development of future ViT-based models for similar image classification applications.
**RQ3**: To answer RQ3, we can maintain that assessing the ViT model’s performance on the BRACS dataset provides a comprehensive examination of its ability to generalize across a spectrum of breast carcinoma sub-types. We obtain a valuable opportunity to rigorously validate the model’s diagnostic accuracy from the dataset’s wide-ranging diversity, including different lesions and tissue samples. This evaluation ensures an understanding of the model’s applicability beyond the specific dataset, enhancing confidence in its broader generalization capabilities.

Based on the ViT model, many researchers have started to propose a range of variants for breast cancer histopathology image classification, as we have previously discussed. Generally, they either utilize an ensemble of ViT models or do not employ the BACH dataset, preferring instead the BreakHis dataset, as highlighted in a recent review by Karuppasamy [40]. Therefore, to the best of our knowledge, our approach is the first one that adopts the base version of ViT on the BACH dataset.

Although the choice to utilize a ViT architecture has shown several advantages for this task, we have identified some limitations. The first one stems from the nature of the BACH dataset. In fact, the challenge organizers have made only accuracy available as the model evaluation metric for the post-challenge phase. Therefore, we have attempted to overcome this issue by generalizing the model through the use of other datasets. The second one arises from the small size of the test set, which consists of 100 unlabeled images. Consequently, we introduce the implementation of 5-fold cross-validation in our study. Hence, potential directions for future work include not only augmenting the dataset through the acquisition of additional breast cancer histopathology images but also developing a pretrained model exclusively trained on breast tissue, similar to HistoEncoder, which is trained on prostate tissue.

## Figures and Tables

**Table 1:** Summary of findings of published breast cancer classification models.

Reference Number	Dataset	Methods	Accuracy
Alotaibi et al. [26]	BreakHis	ViT + DeiT	0.98 (8-class)
Baroni et al. [4]	BACH	ViT	0.91
Brancati et al. [16]	BACH	Resnet-34, 50, 101	0.86
Chennamsetty et al. [9]	BACH	ResNet-101 + DenseNet-161	0.87
He et al. [28]	BreakHis	DecT	0.93
	BACH	DecT	0.79
	UC	DecT	0.81
Kwok [12]	BACH	Inception-Resnet-v2	0.87
Roy et al. [17]	BACH	PBC with CNN	0.87
Sriwastawa and Arul Jothi [29]	BreakHis	MaxViT	0.916
	IDC	MaxViT	0.92
	BreakHis + IDC	MaxViT	0.92
Tummala et al. [24]	BreakHis	Ensemble of SwinTs	0.99 (2-class), 0.96 (8-class)
Wang et al. [22]	BUSI	CT + ViT + ATS	0.95
	BreakHis	CT + ViT + ATS	0.98 (2-class)
Yao et al. [39]	BACH dataset	Ensemble of 5 models	0.92

**Table 2:** Experimental results.

Base Model	Pretrain Strategy	Resize Dimension	Data Augmentation	Tile Overlap	Patch Size	Accuracy Score
ViT	no	no	no	256	32	0.53
ViT	no	224 × 224	no	256	16	0.53
ViT	vit-base	224 × 224	no	256	16	0.84
ViT	vit-base	224 × 224	no	no	16	0.84
ViT	vit-base	224 × 224	Geometric, Color	no	16	0.90
ViT	vit-base	224 × 224	Geometric, Color, Set B	no	16	0.91

**Table 3:** Experimental results: pretraining outcomes.

Base Model	Pretrain Strategy	Data Augmentation	Accuracy Score
ViT	vit-base	Geometric, Color, Set B	0.91
ViT	vit-base	Geometric, Color	0.90
ViT	HistoEncoder prostate_small	Geometric, Color, Set B	0.89
ViT	HistoEncoder prostate_medium	Geometric, Color, Set B	0.86
ViT	vit-base	no	0.84
ViT	vit-base	no	0.84

**Table 4:** Five-fold cross-validation performance metrics for histopathological image classification using a ViT model.

Class	Fold 1	Fold 2	Fold 3	Fold 4	Fold 5	μ	$\sigma^{2}$
**Accuracy **							
Normal	1.00	0.80	0.80	0.60	0.95	0.83	0.16
Benign	0.75	0.65	0.65	0.70	0.80	0.71	0.07
In situ	0.85	0.90	0.65	0.85	0.75	0.80	0.10
Invasive	1.00	0.45	1.00	0.85	0.90	0.84	0.23
**Precision**							
Normal	0.95	0.89	0.89	0.63	0.95	0.86	0.13
Benign	0.83	0.57	0.59	0.64	0.76	0.68	0.11
In situ	0.81	0.75	0.87	0.81	0.83	0.81	0.04
Invasive	1.00	0.60	0.80	0.94	0.86	0.84	0.15
**Recall**							
Normal	1.00	0.80	0.80	0.60	0.95	0.83	0.16
Benign	0.75	0.65	0.65	0.70	0.80	0.71	0.07
In situ	0.85	0.90	0.65	0.85	0.75	0.80	0.10
Invasive	1.00	0.45	1.00	0.85	0.90	0.84	0.23
**F1-Score**							
Normal	0.98	0.84	0.84	0.62	0.95	0.85	0.14
Benign	0.79	0.60	0.62	0.67	0.78	0.69	0.09
In situ	0.83	0.82	0.74	0.83	0.79	0.80	0.04
Invasive	1.00	0.51	0.89	0.89	0.88	0.83	0.19

**Table 5:** BRACS_RoI dataset performance metrics.

	Normal	Benign	In situ	Invasive	Overall
0-N	**0.85**	0.09	0.04	0.02	
1-PB	0.37	**0.36**	0.14	0.13	
2-UDH	0.23	**0.20**	0.50	0.07	
3-FEA	0.12	0.35	0.41	0.12	
4-ADH	0.10	0.12	0.71	0.06	
5-DCIS	0.04	0.06	**0.88**	0.02	
6-IC	0.06	0.03	0.03	**0.88**	
**Accuracy**					0.74

## Data Availability

The data used for the study were derived from the following resources publicly available: Grand Challenge on BreAst Cancer Histology images (BACH) (https://iciar2018-challenge.grand-challenge.org) BReAst Carcinoma Subtyping (BRACS) dataset (https://www.bracs.icar.cnr.it) MSCA European Project AIDPATH (https://aidpath.eu)
